# Nutrient transporter expression in both the placenta and fetal liver are affected by maternal smoking

**DOI:** 10.1016/j.placenta.2019.02.010

**Published:** 2019-03

**Authors:** Natasha Walker, Panagiotis Filis, Peter J. O'Shaughnessy, Michelle Bellingham, Paul A. Fowler

**Affiliations:** a Institute of Medical Sciences, University of Aberdeen, Foresterhill, Aberdeen, AB25 2ZD, UK; b Institute of Biodiversity, Animal Health and Comparative Medicine, College of Medical, Veterinary and Life Sciences, University of Glasgow, Glasgow, G61 1QH, UK

**Keywords:** Human, Placenta, Nutrient transport, Maternal smoking, FAST, Fast Alcohol Screening Test, NHS, National Health Service, SAFeR, Scottish Advanced Fetal Research Study, SLC, solute carrier, SIMD, Scottish Index of Multiple Deprivation

## Abstract

**Introduction:**

The placenta controls nutrient transfer between mother and fetus via membrane transporters. Appropriate transplacental passage of nutrients is essential for fetal growth and development. We investigated whether transporter transcript levels in human placenta-liver pairs from first and early second trimester pregnancies exhibit gestational age- or fetal sex-specific profiles and whether these are dysregulated by maternal smoking.

**Methods:**

In a step-change for the field, paired placenta and fetal livers from 54 electively terminated, normally-progressing pregnancies (7–20 weeks of gestation, Scottish Advanced Fetal Research Study, REC 15/NS/0123) were sexed and cigarette smoking-exposure confirmed. Thirty-six nutrient transporter transcripts were quantified using RT-qPCR.

**Results:**

While fetal, liver and placenta weights were not altered by maternal smoking, levels of transporter transcripts changed with fetal age and sex in the placenta and fetal liver and their trajectories were altered if the mother smoked. Placental levels of glucose uptake transporters *SLC2A1* and *SLC2A3* increased in smoking-exposed fetuses while smoking was associated with altered levels of amino acid and fatty acid transporter genes in both tissues. *SLC7A8*, which exchanges non-essential amino acids in the fetus for essential amino acids from the placenta, was reduced in smoking-exposed placentas while transcript levels of four hepatic fatty acid uptake transporters were also reduced by smoking.

**Discussion:**

This data shows that fetal sex and age and maternal smoking are associated with altered transporter transcript levels. This could influence nutrient transport across the placenta and subsequent uptake by the fetal liver, altering trophic delivery to the growing fetus.

## Highlights

• Nutrient transporters show differential expression in first/second trimesters.
• Maternal smoking alters transporter expression of three essential nutrient groups.
• Fatty acid transporter expression is reduced in smoke-exposed fetal livers.

## 1 Introduction

**Fig. 1:**
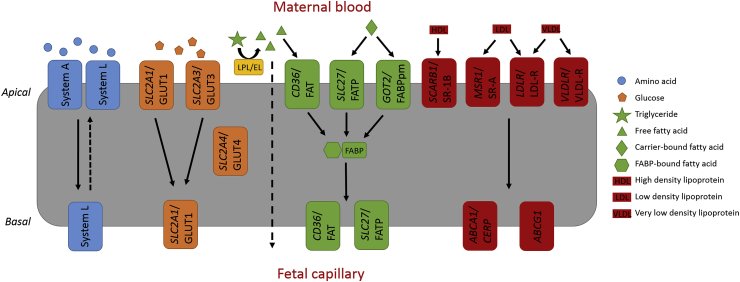
**Schematic summary of transplacental nutrient transport via membrane transport proteins.** Placental nutrient transporters (gene name in italics) with confirmed syncytiotrophoblast location. Uptake of nutrients from maternal circulation occurs on the apical membrane where villous trees are in contact with maternal blood. Transport direction of the main nutrient groups also shown. Non-essential amino acids are taken up by sodium dependent transporters (System A). These non-essential amino acids can be exchanged for essential amino acids by a sodium independent exchanger (System L). Glucose is transported via GLUT1 and GLUT3 and an asymmetrical expression pattern maintains glucose gradient favourable of transport towards fetal capillaries. GLUT4 is stored in syncytial cytoplasm and responsible for increased glucose transport under insulin stimulation in early stages of pregnancy. Maternal TG are converted to FFA by cytoplasm-associated lipases (endothelial and lipophilic) where they are taken up by FATP or FAT then bound to FABP for trafficking towards fetus. FABPpm has high affinity for LCPUFA. FFA can diffuse across syncytiotrophoblast (broken line) but not at an adequate rate to meet fetal requirement. Maternal cholesterol is taken up by SR-1B, SR-A, LDL receptor or VLDL receptor and is believed to exit the syncytiotrophoblast by ABCA1 and ABCG1 (note ABCA1 is also expressed apically). ABC ATP-binding cassette; CERP cholesterol efflux regulatory protein; EL endothelial lipase; FABP fatty acid binding protein; FABPpm plasma membrane fatty acid binding protein; FAT fatty acid translocase; FATP fatty acid transporter protein; FFA free fatty acids; HDL high-density lipoprotein; LDL low-density lipoprotein; LPL lipoprotein lipase; SR scavenger receptor; TG triglycerides; VLDL very low-density lipoprotein.

The placenta ensures appropriate passage of nutrients from the maternal circulation to the fetal circulation. Control of nutrient availability to the fetus is vital and abnormal birthweight is linked to disease incidence later in life [1]. Since fetal nutrient demand is not met by diffusion alone, nutrient availability in the fetal compartment is dependent on appropriate expression profiles of placental membrane transporters in the syncytiotrophoblast and fetal capillaries. Transplacental transfer of amino acids, fatty acids, glucose and cholesterol, essential nutrients for fetal development, is mediated by well-characterised syncytial membrane transporters in the human placenta (Fig. 1). However, information on other placental transporters is sparse and conflicting [2]. Unfortunately, most data available are from term placentas and expression patterns across gestation, particularly in the early second trimester, are inadequately understood. Uptake of nutrients into the fetal liver depends, similarly, on these uptake transporters, although their expression in the fetal liver is also poorly characterised. Characterising these patterns of expression and how they can be changed by maternal factors is important to better understand how modifiable maternal lifestyle can influence placental function and fetal development.

Of the blood supply received by the fetal liver, 70% is directly from the umbilical vein [3] making the liver a checkpoint for distribution of maternally derived nutrients, after the placenta. Our understanding of the functional partnership between the fetal liver and placenta remains to be fully realized, but the concept that it is an interactive relationship is well established [4]. Additionally, the organization of the syncytiotrophoblast is unique to humans, so understanding placental transport and placenta/liver interactions cannot rely only on animal studies.

Maternal lifestyle factors such as smoking [[5], [6], [7]], diet [8] and alcohol consumption [9] during pregnancy are known to influence placental function. Maternal smoking remains the largest avoidable factor for poor pregnancy outcome. Indeed, expression of amino acid transporter genes (*SLC7A5/6*) are known to be altered by pre-pregnancy smoking in term placentas [10]. The impacts of maternal smoking on metabolic enzyme [11] and protein [12] expression in the human fetal liver have been reported, and direct effects of cigarette smoke components demonstrated in cultured hepatoblasts [13]. Data on expression of fetal hepatic nutrient transporters, and the influence of smoking, remains sparse, however. Outlining the relationship between maternal factors, alterations in gene expression and the fetal growth trajectory would be essential information to allow potential windows for intervention to be identified and to allow development of biomarkers for altered fetal growth. Understanding how modifiable maternal factors perturb normal placental function would also give clear guidelines to mothers, which would help to ensure the best chance of a healthy post-natal life for the fetus.

Taking these considerations together, we have, for the first time, examined transporter transcript levels of four major nutrient transporter groups in first and second trimester human placentas and matching fetal livers from the same pregnancies. It is well accepted that maternal smoking harms the fetus but the mechanisms underlying the effect are less well known. The fetal liver plays a central role in fetal physiology as well as being a substantial endocrine organ and it cannot be ignored when investigating the *in utero* impact of smoking exposure on the human fetus. We hypothesize that nutrient transporter transcripts in the placenta and fetal liver will be altered by maternal smoking and this exposure may not affect both organs in the same way.

## 2 Materials & methods

### 2.1 Tissue acquisition

The collection of human fetal material was approved by the National Health Service (NHS) Grampian Research Ethics Committees (REC 15/NS/0123). Women over the age of 16 and with a good understanding of English could consent to take part in the Scottish Advanced Fetal Research (SAFeR) study without any change in treatment. The products of conception (fetus and placenta) from electively terminated, normally-progressing pregnancies (7–20 weeks gestation) were collected within 3 hours of delivery. Fetal measurements were taken e.g. body weight, crown-rump length (CRL), and anogenital distance. Umbilical cords, fetal membranes, maternal decidua and any blood clots were removed from placentas prior to weighing. Each placental sample consisted of 6 full-thickness biopsies which were combined then snap-frozen and pulverized in liquid nitrogen to form a representative powder. Fetal livers were removed whole and weighed after the gall bladder was removed (where possible). A 30 mg sample was taken from the main/right lobe of the liver and snap-frozen. All tissues were stored at −80 °C.

### 2.2 Maternal and fetal data

In order to preserve maternal anonymity, maternal health information (BMI, medication etc) routinely collected by independent pregnancy nurse specialists was entered onto study-specific anonymised forms. The nurses sent maternal data to NHS Grampian Data Safe Haven (DaSH) and anonymous Scottish Index of Multiple Deprivation (SIMD) scores (as vigintiles) were returned to the research team. SIMD uses 7 criteria: income (28%), employment (28%), health (14%), education, skills and training (14%), housing (2%), access to services (9%) and crime (5%) to estimate level of deprivation on a scale of 0–20 [14]. The cohort described here are equally spread across this scale. Patients were asked to complete the Fast Alcohol Screening Test (FAST) [15]. This is a validated questionnaire which assesses amount and frequency of alcohol intake. Fetal gestation was calculated from measurements of fetal foot-length taken by ultrasound scan prior to termination of pregnancy. Maternal and fetal information is summarised in Table 1.

**Table 1:** Placentas and fetuses collected between 7 and 20 weeks of gestation.

	SAFeR cohort			Placenta-liver pair qPCR cohort		
	Control (n = 113)	Smoking-exposed (n = 64)	P-value	Control (n = 33)	Smoking-exposed (n = 21)	P-value
Maternal age	27.3 ± 0.7	25.9 ± 0.8	0.23	25.8 ± 1.3	25.1 ± 0.9	0.69
Maternal BMI	26.7 ± 0.6	26.2 ± 0.6	0.56	27.8 ± 1.4	24.6 ± 1	0.10
FAST score	1.5 ± 0.1	1.6 ± 0.2	0.59	1.8 ± 0.3	1.1 ± 0.2	0.08
SIMD score	12.6 ± 0.5	11.5 ± 0.6	0.17	11.1 ± 0.9	11.6 ± 1	0.76
Placenta weight (g)	33.2 ± 3.2	29.1 ± 3.2	0.40	44.3 ± 7.5	29.3 ± 4.2	0.12
Cotinine (ng/g)	**0.02 ± 0.01**	**110.5 ± 15.5**	< 0.001***	**0.1 ** ± **0.1**	**113.4 ± 16.7**	< 0.001***
Fetal age	10.9 ± 0.3	10.9 ± 0.4	0.78	12 ± 0.6	11.2 ± 0.6	0.37
Fetal sex	F = 50 M = 55	F = 29 M = 34	n/a	F = 16 M = 15	F = 13 M = 10	n/a
Fetal weight (g)	30.9 ± 5.6	21.5 ± 4.8	0.24	45.4 ± 12	24.8 ± 9.2	0.21
Liver weight (g)	1.5 ± 0.3	1.2 ± 0.3	0.44	2 ± 0.5	1.1 ± 0.4	0.19
CRL (mm)	62.6 ± 4.5	61 ± 5.4	0.82	71.6 ± 8.9	56.8 ± 8.1	0.25
Ponderal index (mg/mm^3^)	0.1 ± 0.003	0.1 ± 0.003	0.31	0.1 ± 0.01	0.1 ± 0.004	0.35

No significant differences were seen in maternal and fetal characteristics between smoking-exposed and control groups in both SAFeR (to date) and placenta-liver pair (current study) cohorts except cotinine level, shown in bold. *P*-values derived from t-tests. FAST total score indicates frequency and severity of alcohol intake. SIMD low numbers indicate more deprived areas. BMI: body mass index (kg/m^2^); CRL: crown-rump length; F: female; FAST: fast alcohol screening test; M: male; SIMD: Scottish Index of Multiple Deprivation. Ponderal index is an index of fetal leanness. Values shown as mean ± SEM.

### 2.3 Transcript and cotinine analysis

54 paired placenta and liver samples from the same fetus were sexed (by presence/absence of Y-specific gene, *SRY*) and smoke exposure validated by measurement of placental cotinine, an active metabolite of nicotine, using LC-MS/MS (Supplementary Method 1). The cotinine range for non-smokers was <5 ng/g wet tissue weight and for smokers it was ≥10 ng/g. DNA, RNA and protein were extracted simultaneously from tissues (∼30 mg) using Qiagen Mini-prep kits (Qiagen Ltd), including a DNAse step (Qiagen RNase-Free DNase Set) according to manufacturer's guidelines. RNA was reverse transcribed using a GoScript Reverse Transcriptase kit (Promega). For quantitative real-time PCR (RT-qPCR), primers were designed to amplify nutrient transporter transcripts using Primer-BLAST (https://www.ncbi.nlm.nih.gov/tools/primer-blast/) (Table S1), with criteria set as previously described [16]. Transcript expression was quantified by RT-qPCR (LightCycler 480 SYBR Green I Master, Roche) (Roche LightCycler 480). Samples were run in duplicate against a 6 point standard curve for each transcript (Absolute Quantification analysis) using parameters; 95 °C for 5 min, 45 cycles of 95 °C for 15 s, 63 °C for 15 s and 72 °C for 15 s followed by a melting curve. A selection of samples were repeated across plates to test for inter-plate variation. To identify appropriate house-keeping genes (HKG) for these studies; data from 7 (placenta) and 4 (liver) candidate HKG were compared using NormFinder.xla Excel add-in (Table S2) to check stability across gestational age, fetal sex and smoking-exposure. Succinate dehydrogenase A (*SDHA*) was the most stable in both placenta and fetal liver (both M = 0.004). Normalised cycle threshold values for each gene (mean ± SEM) can be found in Supplementary Table S3.

### 2.4 Statistical analysis

Statistical analyses were performed in R statistical software version 3.5.1 using our established linear modelling approach [17] with fetal age (continuous), fetal sex (categorical) and smoking-exposure (categorical, confirmed by cotinine), as covariates both singly and in combination. To ensure that maternal alcohol use did not interact with maternal smoking, we included FAST score in our linear model (FAST score + age*sex*smoking-exposure). Significance was accepted at *P* < 0.05.

## 3 Results

Cotinine concentrations correlated closely between the fetus (represented by a fetal liver sample) and placenta (R-squared = 0.82, *P* < 0.001, Supplementary Fig. S1). There were no differences in fetal, placental and liver weights; crown-rump length (CRL); or ponderal index (fetal weight/CRL^3^) between smoking-exposed and control fetuses in the SAFeR cohort (Table 1). Maternal factors, such as alcohol use, were not significantly different between smokers and non-smokers (Supplementary Fig. S2). Time elapsed between delivery to processing of tissue did not affect results (Supplementary Fig. S3).

### 3.1 Developmental ontogeny of nutrient transporter transcripts

Levels of transcripts encoding twenty-two transporters changed across gestational age in the placenta, with the majority (16/22) increasing with advancing gestation. Conversely, levels of 18/36 transcripts were altered by fetal age in the fetal livers, with the majority decreasing (10/18) with advancing gestation. There was consensus in the changing transcript levels between the placenta and liver for *SLC3A2, SLC27A6* and *GOT2* (all ↓) and for *SLC2A4*, *SLC38A2* and *CD36* (all ↑) (Table 2).

**Table 2:** Gestational expression patterns of nutrient transporter transcripts.

Gene	Substrate	Placenta	Liver	Gene	Substrate	Placenta	Liver
*SLC7A5*	neutral AA, thyroid hormone	↓ *	↑*	*SLC2A1*	glucose uptake	**-**	**-**
*SLC7A8*	neutral AA, thyroid hormone	**-**	↑***	*SLC2A3*	glucose uptake/export	**-**	**-**
*SLC43A1*	branched chain AA, phenylalanine	↑***	**-**	*SLC2A4*	glucose uptake early pregnancy (insulin sensitive)	↑***	↑***
*SLC42A2*	neutral AA	**-**	↓***	*SLC2A9*	glucose uptake	**-**	↑*
*SLC1A4*	alanine, serine, cysteine, threonine	↑***	↓*	*SLC27A1*	Long chain fatty acids	↑*	**-**
*SLC1A5*	neutral and zwitterionic AA	↑ **	**-**	*SLC27A2*	Long chain fatty acids/FA metabolism	**-**	**-**
*SLC7A7*	neutral and zwitterionic AA	↑ ***	↓ ***	*SLC27A3*	Long chain fatty acids	**-**	↓**
*SLC7A6*	arginine, leucine and glutamine	**-**	↓***	*SLC27A4*	Long chain fatty acids	**-**	↓**
*SLC3A2*	chaperone	↓**	↓***	*SLC27A6*	Long chain fatty acids	↓***	↓**
*SLC1A3*	l-glutamate, L/d-aspartate	↑***	↓**	*GOT2*	Long chain fatty acids	↓*	↓**
*SLC1A2*	l-glutamate, L/d-aspartate	↓***	↑***	*CD36*	Long chain fatty acids	↑***	↑*
*SLC1A1*	l-glutamate, L/d-aspartate, l-cysteine	↑***	**-**	*MSR1*	Low-density lipoproteins	↑***	**-**
*SLC1A6*	l-glutamate, L/d-aspartate	↑*	**-**	*SCARB1*	High-density lipoproteins	↑*	**-**
*SLC1A7*	l-glutamate	**-**	**-**	*LDLR*	Low and very low-density lipoproteins	**-**	↑**
*SLC38A1*	Glutamine, sodium ions	↓ *	**-**	*VLDR*	Very low-density lipoproteins	**-**	**-**
*SLC38A2*	neutral AA, sodium ions	↑***	↑***	*ABCA1*	Cholesterol and phospholipids	↑*	**-**
*SLC38A4*	neutral AA, preference for alanine	**-**	**-**	*ABCG1*	Cholesterol and phospholipids	↑***	**-**
*SLC16A10*	aromatic acids	↑***	**-**				
*SLC6A6*	taurine, beta-alanine	**-**	**-**				

Ontogeny of transcript expression in human placenta and fetal liver pairs normalised against *SDHA*. Substrate of corresponding membrane transporter given (source:UniProt). AA amino acids; ¶ transcripts encode subunits of a heterodimeric transporter; **↑** increases with gestational age; **↓** decreases with gestational age; - stable across gestation. *P < 0.05 **P < 0.01 ***P < 0.001.

### 3.2 Effects of maternal smoking on nutrient transporter levels in the placenta

**Fig. 2:**
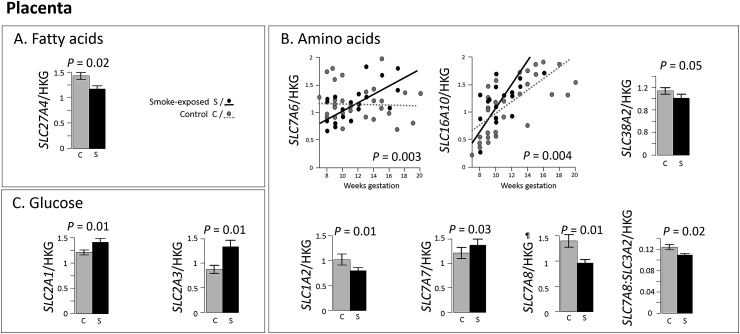
**Maternal smoking is associated with changes in expression levels and gestational expression patterns of placental nutrient transporter transcripts.** Maternal smoking altered levels (shown as histograms) and gestational age patterns (shown as age plots) of transcripts coding for membrane transporters involved in transport of; fatty acids (A), amino acids (B), and glucose (C) in the human placenta. Transporter transcripts that were not changed by fetal sex and transcripts showing no smoking effects are not shown. All data was normalised to *SDHA*. Histograms show mean ± SEM and age plots multi-variate regression using the linear model (*transcript* ∼ age*smoking-exposure). n = 54 placentas, per group: n = 33 controls and n = 21 smoking-exposed. ¶ Transcript encodes subunit of a heterodimeric transporter protein.

Transporter transcript levels were affected by smoking-exposure in the placenta (Fig. 2). *SLC27A4* was decreased compared with controls when the mother smoked (Fig. 2A, *P* = 0.02) and was associated with a greater rate of increase in *SLC7A6* (*P* = 0.003), and *SLC16A10* (*P* = 0.002) levels during gestation (Fig. 2B). A further four amino acid transporter transcripts were significantly influenced by smoking with *SLC7A7* increasing (*P* = 0.03) and *SLC38A2* (*P* = 0.05), *SLC1A2* (*P* = 0.01) and *SLC7A8* (*P* = 0.01) decreasing*. SLC7A8* encodes one subunit of a heterodimer, and its ratio with the second subunit, *SLC3A2*, showed the same association with smoking (*P* = 0.02). Transcripts encoding two major placental glucose uptake transporters (*SLC2A1* and *SLC2A3*) were upregulated in smoking-exposed placentas (Fig. 2C, both *P* = 0.01). Results from studies examining the effects of smoking and gestational age have been compared to relevant literature from 1st, 2nd and term placenta in Table 3.

**Table 3:** Comparison of SAFeR placental results to relevant literature.

gene	covariate	SAFeR (n = 54)	other studies	trimester	sample size	references	Comments
**AMINO ACID TRANSPORTER TRANSCRIPTS**							
*SLC7A5*	age	**↓**	**↑**	Early 2nd vs term	6	[38]	protein
			**↑**	Early 2nd vs term	23	[39]	
	smoking	**-**	**-**	term	102	[10]	
*SLC7A8*	age	**-**	**-**	All	23 (2nd & term) 8 (1st & term	[39]	
	smoking	**↓**	**↑**	term	102	[10]	pre-pregnancy smoking
*SLC43A1*	age	**↑**	**↑**	term	102	[40]	↑ with fetal growth indices, not fetal age
			**-**	All	23 (2nd & term) 8 (1st & term	[39]	
	smoking	**-**	**-**	term	102	[10]	
*SLC42A2*	age	**-**					No relevant study found
	smoking	**-**	**-**	term	102	[10]	
*SLC1A4*	age	**↑**	**↓**	Early 2nd vs term	23	[39]	
	smoking	**-**	**-**	term	102	[10]	
*SLC1A5*	age	**↑**	**-**	Pre-term (34.8) vs term	20	[41]	Protein, small age range
			**-**	All	23 (2nd & term) 8 (1st & term	[39]	
	smoking	**-**	**-**	term	102	[10]	
*SLC7A7*	age	**↑**	**-**	Pre-term (33.8) vs term (38.8)	9	[42]	Protein, small age range
			**↓**	Early 2nd & term	23	[39]	
	smoking	**↑**	**-**	term	102	[10]	pre-pregnancy smoking
*SLC7A6*	age	**-**	**-**	All 3	23 (2nd & term) 8 (1st & term	[39]	
	smoking	**↑**age	**↑**	term	102	[10]	pregnancy smoking
*SLC3A2*	age	**↓**	**-**	Pre-term (34.8 wks) vs term	20	[41]	Protein, small age range
			**-**	All 3	23 (2nd & term) 8 (1st & term	[39]	
	smoking	**-**	**-**	term	102	[10]	
*SLC1A3*	age	**↑**	**↓**	Early 2nd vs term	23	[39]	
	smoking	**-**	**-**	term	102	[10]	
*SLC1A2*	age	**↓**	**↓**	1st vs term	8	[39]	
	smoking	**↓**	**-**	term	102	[10]	
*SLC1A1*	age	**↑**	**-**	All	23 (2nd & term) 8 (1st & term)	[39]	
	smoking	**-**	**-**	term	102	[10]	
*SLC1A6*	age	**↑**	**-**	All	23 (2nd & term) 8 (1st & term)	[39]	
	smoking	**-**	**-**	term	102	[10]	
*SLC1A7*	age	**-**	**-**	All	23 (2nd & term) 8 (1st & term)	[39]	
	smoking	**-**	**-**	term	102	[10]	
*SLC38A1*	age	**↓**	**-**	1st vs term	56	[43]	
			**-**	All	23 (2nd & term) 8 (1st & term)	[39]	
	smoking	**-**	**-**	term	102	[10]	
*SLC38A2*	age	**↑**	**↑**	All	23 (2nd & term) 8 (1st & term)	[39]	
			**-**	1st vs term	56	[43]	
	smoking	**↓**	**-**	term	102	[10]	
*SLC38A4*	age	**-**	**-**	All	23 (2nd & term) 8 (1st & term)	[39]	
			**↓**	1st vs term	56	[43]	
	smoking	**-**	**-**	term	102	[10]	
*SLC16A10*	age	**↑**	**-**	All	23 (2nd & term) 8 (1st & term)	[39]	
	smoking	**↑**age	**-**	term	102	[10]	pre-pregnancy smoking
*SLC6A6*	age	**-**	**-**	1st vs term	56	[44]	Transcript and protein agreed
	smoking	**-**	**-**	term	61	[28]	TauT activity, not expression, few smokers (n = 5)
**GLUCOSE TRANSPORTER TRANSCRIPTS**							
*SLC2A1*	age	**-**	**↑**	1st vs term	45	[32]	protein
	smoking	**↑**					No relevant study found
*SLC2A3*	age	**-**	**↓**	All	22	[33]	protein
	smoking	**↑**					No relevant study found
*SLC2A4*	age	**↑**	**↓**	1st vs term	45	[32]	Transcript & protein
	smoking	**-**					No relevant study found
*SLC2A9*	age	**-**					No relevant study found
	smoking	**-**					No relevant study found
**FATTY ACID TRANSPORTER TRANSCRIPTS**							
*SLC27A1*	age	**↑**					No relevant study found
	smoking	**-**					No relevant study found
*SLC27A2*	age	**-**					No relevant study found
	smoking	**-**					No relevant study found
*SLC27A3*	age	**-**					No relevant study found
	smoking	**-**					No relevant study found
*SLC27A4*	age	**-**					No relevant study found
	smoking	**↓**					No relevant study found
*SLC27A6*	age	**↓**					No relevant study found
	smoking	**-**					No relevant study found
*GOT2*	age	**↓**					No relevant study found
	smoking	**-**	**↑**	term	102	[10]	Pre-pregnancy smoking
*CD36*	age	**↑**					No relevant study found
	smoking	**-**					No relevant study found
**CHOLESTEROL/LIPID TRANSPORTER TRANSCRIPTS**							
*MSR1*	age	**↑**					No relevant study found
	smoking	**-**					No relevant study found
*SCARB1*	age	**↑**	**↓**	1st vs term	Cell culture	[45]	mRNA and protein agreed
	smoking	**-**					No relevant study found
*LDLR*	age	**-**	**↓**	All	26	[46]	
	smoking	**-**	**↓**	term	Cell culture	[36] [37]	Cadmium only, *in vitro*
*VLDLR*	age	**-**	**↑**	1st vs term	11	[47]	Small n, term placentas were laboured
	smoking	**-**					No relevant study found
*ABCA1*	age	**↑**	**↓**	Pre-term (32.8 wks) vs term (39.1 wks)	29	[48]	
			**-**	1^st^ vs term	48	[49]	Transcript and protein
			**↑**	1^st^ vs term	20	[50]	1^st^ trimester only 7–9 wks
	smoking	**-**					No relevant study found
*ABCG1*	age	**↑**	**-**	Pre-term (32.8 wks) vs term (39.1 wks)	26	[48]	Transcript and protein
	smoking	**-**					No relevant study found

### 3.3 Effect of maternal smoking on nutrient transporter transcript levels in the fetal liver

**Fig. 3:**
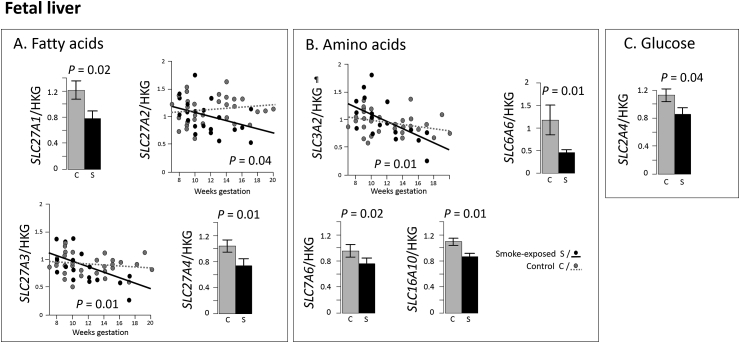
Maternal smoking is associated with changes in expression levels and gestational expression patterns of nutrient transporter transcripts in the fetal liver. Maternal smoking altered levels (shown as histograms) and gestational age patterns (shown as age plots) of transcripts coding for membrane transporters involved in transport of; fatty acids (A), amino acids (B) and glucose (C) in the human fetal liver. Transporter transcripts that were not changed by fetal sex and transcripts showing no smoking effects are not shown. All data was normalised to *SDHA*. Histograms show mean ± SEM and age plots multi-variate regression using the linear model (*transcript* ∼ age*smoking-exposure). n = 54 placentas, per group: n = 33 controls and n = 21 smoking-exposed. ¶ Transcript encodes for subunit of a heterodimeric transporter protein.

All placental nutrient transporters quantified were also expressed in the fetal liver. Smoking-exposure was associated with changes to transcripts involved in fatty acid, amino acid and glucose trafficking (Fig. 3). Hepatic levels of two fatty acid transporters (*SLC27A1* and *SLC27A4*, Fig. 3A), three amino acid transporters (*SLC6A6, SLC7A6* and *SLC16A10,* Fig. 3B), and a glucose transporter (*SLC2A4*, Fig. 3C) were all decreased (*P* < 0.05) in smoking-exposed fetuses. Smoking-exposure was associated with decreased *SLC27A2* transcript levels while controls remained stable with fetal age. Levels of *SLC27A3* and *SLC3A2* decreased with age in control fetuses and the rate of this decline was accelerated by maternal smoking (both *P* = 0.01). *SLC3A2* encodes a chaperone protein required for function of LAT1 and LAT2 (encoded by *SLC7A5*, *SLC7A8* respectively) but these transcripts were unaffected by smoking-exposure in the fetal liver.

### 3.4 Effect of fetal sex on nutrient transporter transcript expression

Some transporters exhibited sex-specific patterns of transcript levels across the fetal age range analysed (Supplementary Fig. S4). In the fetal liver, levels of *SLC7A8* and *SLC1A7* were decreased in males compared to females (*P* = 0.04, *P* = 0.05 respectively) while, in the placenta, *SLC27A6* was increased in males compared to females (*P* = 0.04).

## 4 Discussion

In this study we have characterised transcript profiles of a large panel of key nutrient transporters in normal human first and second trimester placentas and paired fetal livers from the same pregnancies. The placenta is the route of delivery of nutrients to the fetus while the fetal liver is one of the initial fetal organs at which these nutrients can be distributed to the rest of the fetus. Studying both organs simultaneously from the same fetus in the first and second trimester of human pregnancy represents a first in the field. Our results show that maternal smoking is associated with significant changes in transcript levels for some of the key nutrient transporters studied. This may, therefore, be a fundamental way in which maternal smoking could affect eventual transporter activity with knock-on effects on fetal growth and development and longer-term effects postnatally.

The current study is limited to investigation of mRNA levels and takes no account of translational or post-translational modifications. The results need to be treated with suitable caution, therefore, and more work is needed to fully understand the effects of maternal lifestyle on transporter activity and consequent nutrient allocation to the fetus. Nevertheless, alterations in transcript expression are a recognised way in which changes in cellular activity can occur and this represents a first step towards understanding how maternal smoking affects nutrient transport in the first and second trimesters. In addition, differential methylation of genes occurs in the placentas of smokers compared to non-smokers [18,19] which offers a potential mechanism for how smoking can cause changes in gene expression. There is evidence gene expression can differ between the lobes of the liver in fetal baboons [20]. However, we believe there was no accounting for False Discovery Rates and a significant proportion of these gene differences could be due to higher levels of white blood cells and lower levels of hepatocytes. The liver samples in the current study were taken from the right lobe as it is the larger lobe and 30 mg tissue can be taken at all gestational ages to ensure consistent downstream analysis. Whether there are significant gene expression pattern differences between hepatic lobes in the human fetal liver is subject to studies beyond the scope of the current manuscript.

It is well documented that women who smoke during pregnancy are more likely to have a neonate which is small for gestational age [21]. Given that ultrasound in the first and second trimester fails to predict growth restriction [22] and smoking effects on fetal size are mainly seen in the third trimester [23], it was unsurprising that we did not see any gross morphological differences in smoking-exposed fetuses. The placenta undergoes structural changes throughout pregnancy and maternal smoking has been shown to affect villous structure [24]. Thus, it is possible that changes in cellular composition in response to maternal smoking could contribute to the observed changes in transcript levels in the current study.

Those transporter transcripts which increase towards the end of the second trimester in liver and placenta are likely to reflect the rise in fetal demand for those nutrients. Similarly, those transporters that decrease over the same period may reflect either less of a need for the substrate, or less of a need to source the substrate maternally. For nine genes, the developmental trajectories matched what is seen at term (Table 3). Where both placental and liver transcript patterns change, more than half were in the same direction as fetal age which suggests a hepatic response to changing nutrient efflux from the placenta.

Previous studies have tried to characterise amino acid transporter expression across gestation. These trajectories are often contradictory, as demonstrated by the comparisons between this study and others (Table 3). Nevertheless, seven amino acid transporter transcripts displayed age patterns which agree with other studies and often match what is seen at term (i.e. *SLC1A2*). When examining expression of individual transporters, our findings generally agreed with Day and colleagues [10]. *SLC7A6* was increased by smoking in both studies and where we did not see an association with maternal smoking, neither did they. Conversely, some findings differed (*SLC7A7*, *SLC7A8* and *SLC16A10*) but were only significantly associated with pre-pregnancy smoking rather than smoking exposure *in utero*. Maternal smoking has been reported to depress amino acid transport activity in the placenta [25] but the number of placentas studied was not large (a quarter of our cohort) and some were laboured which could alter transport activity and smoking-exposure was based on maternal admission only, missing any second-hand exposure.

*SLC7A8,* along with *SLC3A2,* encode a heterodimeric exchanger which localises to both sides of the plasma membrane and trades non-essential amino acids for essential amino acids from the syncytial cytoplasm [26], directing them towards the fetus. Decreased levels may reflect disruption of this process in smoking-exposed placentas. Due to the broad substrate profile of *SLC7A8*, transfer of several amino acids may be affected. TauT/*SLC6A6* transports taurine which is thought to be essential for development since it is the most abundant amino acid in the placenta and also has cytoprotective functions [27]. Levels of *SLC6A6* were not associated with maternal smoking in our 1st and early 2nd trimester placentas and Ditchfield *et al.* [28] demonstrate that TauT activity is not influenced by maternal smoking at term. Nevertheless, a smoking-associated decrease of *SLC6A6* was seen in fetal livers in the current study which may affect hepatic uptake of taurine.

It is thought that the main source of fetal glucose is maternally derived [29,30]. *SLC2A1* and *SLC2A3* encode well characterised glucose-uptake transporters in the placenta and, if the smoking effects found in the current study are translated to functional protein, the transplacental glucose gradient could be affected as this is dependent on asymmetrical distribution of transporters on the plasma membrane (Fig. 1). GLUT4 (encoded by *SLC2A4*) is believed to take up 50% of the body's glucose [31] and we show lower *SLC2A4* levels in the fetal liver if the mother smoked. Such alterations to glucose availability could lead to fetal hyper/hypoglycaemia. Smoking-exposure literature was scarce for *SLC2A*/GLUT transporters (Table 3) and gestational trajectories found in the current study did not agree well with protein studies from other literature [32,33]. Our results are the first to show an association between maternal smoking and GLUT mRNA expression in the human fetus and its placenta.

Fatty acids are the highest energy requirement of the fetus [34]. *SLC27A4/*FATP4 is the most abundant long chain fatty acid uptake transporter and the smoking-induced change could have implications for transplacental movement of fatty acids. Four of the seven fatty acid transporters studied reduced in the liver by smoking and if this mRNA finding translates to functional protein; reduced hepatic fatty acid uptake could be a consequence. Day *et al.* [10] have reported increased *GOT2* in placentas where the mother was a former smoker but we saw no association with pregnancy smoking. In an *ex vivo* perfusion model of the term placenta, fatty acid transport activity was reported to be unaffected by maternal smoking [35] albeit with a small sample size (n = 10). There are no other studies of gestational expression or smoking-exposure effects on fatty acid transporters in the human placenta.

Smoking-exposure did not alter the expression levels of *MSR2, SCARB1, LDLR, VLDLR, ABCA1 or ABCG1* in either the placenta or the fetal liver in this study. Two previous studies [36,37] have shown that cadmium, a component of cigarette smoke, is associated with a reduction in expression of the LDL receptor *in vitro*. We did not see an association with exposure to maternal smoking and *LDLR* (*P* = 0.69). It is likely, therefore, that the levels of cadmium reaching the placenta/fetal liver *in vivo* do not reach those used *in vitro*.

In conclusion, our study of paired placenta and livers from the same, normally progressing human pregnancy has characterised expression patterns of key nutrient transporters across a wide pregnancy window, from 7 to 20 weeks gestation. We report that cigarette smoking, a modifiable maternal habit, is associated with changes in transcript level of nine nutrient transporters in the placenta (eight in the matching livers). These findings highlight a vulnerability for the fetus, especially in the placenta as changes in transporter function are likely to alter nutrient delivery to the fetal compartment and adversely impact on overall fetal growth and development.

## Sources of support

This work was supported by Glasgow Children's Hospital Charity: YRSS/PHD/2016/05, to NW and UK Medical Research Council: MR/L010011/1, to PAF & PJOS and MR/P011535/1 to PAF. Funders and authors NW, PF, PJOS, MB and PAF have no conflicts of interest.

## Declarations of interest

None.

## Appendix A Supplementary data

Supplementary material_Jan 2019_clean_V2 Supplementary material_Jan 2019_clean_V2
