# Supplementary material for: Nutrient transporter expression in both the placenta and fetal liver are affected by maternal smoking
Source: Placenta. 2019 Mar;78:10–7. doi: 10.1016/j.placenta.2019.02.010 (PMC6461130; doi:10.1016/j.placenta.2019.02.010)
Supplement: Supplementary material_Jan 2019_clean_V2 [file mmc1.docx]

| **Supplementary Table S1. Primer sequences** | | | | |
| --- | --- | --- | --- | --- |
|  | **Protein** | **Gene** | **Forward sequence** | **Reverse sequence** |
| **Amino acid transporters** | LAT1 | *SLC7A5* | GATCAACCCCTACAGAAACCTGCCC | GACATGACGCCCAGGTGATAGTTCC |
|  | LAT2 | *SLC7A8* | GCCTGGATCATGCCCATTTCTGTTG | GTGGAGATGCATGTGAAGAGCAGGG |
|  | LAT3 | *SLC43A1* | CATGGCTGCTGTGAACAAGATGCTG | GTAGAACCCAACTGTCTCTGCCACC |
|  | LAT4 | *SLC42A2* | GAGATGGCCTGGAGAGTGTGTTTCC | GTCCAGAAAGGGTAGCTTCAGCAGG |
|  | ASCT1 | *SLC1A4* | CATTTGCTACCTGCTCCAGCTCA | CTGCTGATCCTCTTGTCCACACCAT |
|  | ASCT2 | *SLC1A5* | ATCATCACCATCCTGGTCACGGC | CCCAGAGCGTCACCTTCTACATTGAG |
|  | y+LAT1 | *SLC7A7* | TTCCCTTTTACTGCTGGCTGGGAAG | GCATTGCCTCCTTGGTCCTGGATA |
|  | y+LAT2 | *SLC7A6* | GATGCTGTGGCTGTGACATTTGCTG | ATCATGGACAGAAGGTCCGGTAGGT |
|  | CD98 | *SLC3A2* | AACATGACTGTGAAGGGCCAGAGTG | GGTCCCAGTGGCGGATATAGGAGAA |
|  | EAAT1 | *SLC1A3* | CCTTGGATTTACCCTCCGACCATACA | TATCTAGCGCCGCCATTCCTGTG |
|  | EAAT2 | *SLC1A2* | AGGCCAAGCTGATGGTGGATTTCTT | GATACCCAGGGGAGAGTACCACATGA |
|  | EAAT3 | *SLC1A1* | TCGAGAACACAGCAACCTCTCAACT | GTGCAGCAACACCTGTAATCATGCTG |
|  | EAAT4 | *SLC1A6* | TGCTTCAAACAGTTCAAGACGCAGT | ACGGGTACAGTCTCCTCAAAGCTCA |
|  | EAAT5 | *SLC1A7* | TCTCATGCGTGGAATGGTGCTGTG | GTAACTAATTTCCTGTGGTGAGAGGCG |
|  | SNAT1 | *SLC38A1* | GCCAGCGGAAGGAACAGGAAGAAG | ATGGAAGCTTGACACCCCTGTTAGC |
|  | SNAT2 | *SLC38A2* | CTGAAAGACCGCAGCCGTAGAAGAA | ACACAGCCAGACGGACAATGAGAAG |
|  | SNAT4 | *SLC38A4* | GGACACCCCACTCACACAGAACAG | ACTGCTGCTCTCATCATCTGGTTCG |
|  | TAT1 | *SLC16A10* | TGGTGCAACGGGTCGGTGTT | TGGAGAGAGAACCTACCCATGCTGT |
|  | TauT | *SLC6A6* | GATCGACTTGTGCTCTCTGTGGCT | AGGCCGCTCCCAAACAGGAAAATAA |
| **Glucose transporters** | GLUT1 | *SLC2A1* | CTGTCTGGCATCAACGCTGTCTTCT | GATACCGGAGCCAATGGTGGCATAC |
|  | GLUT3 | *SLC2A3* | GCGAGACCCAGAGATGCTGTAATGG | TATCAGAGCTGGGGTGACCTTCTGT |
|  | GLUT4 | *SLC2A4* | GGTTTCCAGTATGTTGCGGAGGCTA | AGGTGAAGATGAAGAAGCCCAGCAG |
|  | GLUT9 | *SLC2A9* | GACTGTGTCCATATTCGCCATCGGT | ATCTATGCCCATGATGAAGCGTCCC |
| **Fatty acid transporters** | FATP1 | *SLC27A1* | TCAGCTCTCTCTGCTTCCCCAG | AGAGACCGAAGAGGTCTCGCCT |
|  | FATP2 | *SLC27A2* | TGGCATTCACGGATGTATTGTGGCT | ACGGTCATTTGGTTTCTGTGGTGAGT |
|  | FATP3 | *SLC27A3* | TTTTCTTCAACACTGGGGACCTGCT | CCACATTCTCCCCCTTCCACCTGA |
|  | FATP4 | *SLC27A4* | TACCACTCAGCAGGAAACATCGTGG | GGTAGCGGCACAGTTCACCAATGTA |
|  | FATP6 | *SLC27A6* | TGAGTTGGGTGCCACTTGTGTGTTA | CCATTTCCAATTGCCAAACGCACCT |
|  | FABPpm | *GOT2* | CCTAGCGTCCGCAAGTTTGTCACT | TCCCTGAAGATGGGTGTGTGGTTTC |
|  | FAT | *CD36* | AGAAAAATGGGCTGTGACCGGAACT | ACCTTCTTCGAGGACAACTTGCTTTT |
| **Cholesterol/lipid transporters** | SR-A | *MSR1* | CCTCGTGTTTGCAGTTCTCATCCCT | CGCTGTCATTTCCTTTTCCCGTGAG |
|  | SR-1B | *SCARB1* | CACTCCTTGTTCCTGGACATCCACC | AATGTGTGAAGAGTCTCCCCCTCCA |
|  | LDL-R | *LDLR* | CTGTCTCTGTTGCGGATACCAAGGG | TCAGTCACCAGCGAGTAGATGTCCA |
|  | VLDL-R | *VLDLR* | TGGTCGCTGTATTACGCTGTTGTGG | CATCACACTTCCATCGGCTGGGAAC |
|  | CERP | *ABCA1* | GAGACGCAAACACAAAAGTGGAAAACAG | AGCAGCAGCTGACATGTTTGTCTT |
|  | ABCG1 | *ABCG1* | TTCTTCGTCAGCTTCGACACCATCC | GCCATAGATGGAGAGGATGACCCCT |

Primer pairs were designed in PRIMERBLAST to PCR cycle specifications and spanning exon-exon junctions. PCR gels were electrophoresed to ensure that the correct product size was amplified and to ensure that non-specific amplification with genomic DNA was not occurring. Reaction void of reverse transcriptase and H_2_O were used as controls (not shown).

| **Supplementary Table S2. Selection of Candidate house-keeping genes (HKG)** | |
| --- | --- |
|  | **Stability value (M)** |
| **PLACENTA** | |
| *TOP1* | 0.011 |
| *YWHAZ* | 0.008 |
| *SDHA* | 0.004 |
| *B2M* | 0.011 |
| *PMM1* | 0.005 |
| *SFRS4* | 0.004 |
| *UBC* | 0.008 |
| **LIVER** | |
| *SDHA* | 0.004 |
| *SRFS4* | 0.009 |
| *B2M* | 0.005 |
| *UBC* | 0.003 |

For each transcript the data was analyzed using NormFinder and stability values calculated across all groups (control male, control female, smoke-exposed male, smoke-exposed female). SDHA was used as the HKG for the current study based on stability in both the placenta and the liver.

| **Supplementary Table S3.** Average cycle threshold (ct) values (normalized against SDHA) for all amplicons | | |
| --- | --- | --- |
| **Amplified gene** | **Ct placenta** (mean±SEM) | **Ct liver**  (mean±SEM) |
| *SLC7A5* | 0.979±0.004 | 0.976±0.003 |
| *SLC7A8* | 1.030±0.004 | 0.933±0.004 |
| *SLC43A1* | 1.182±0.005 | 0.952±0.004 |
| *SLC42A2* | 0.199±0.076 | 1.092±0.007 |
| *SLC1A4* | 1.142±0.004 | 1.043±0.004 |
| *SLC1A5* | 1.024±0.002 | 0.963±0.004 |
| *SLC7A7* | 1.184±0.004 | 1.199±0.005 |
| *SLC7A6* | 1.070±0.003 | 1.190±0.005 |
| *SLC3A2* | 0.974±0.003 | 0.989±0.005 |
| *SLC1A3* | 1.071±0.002 | 1.340±0.008 |
| *SLC1A2* | 1.101±0.005 | 1.139±0.007 |
| *SLC1A1* | 1.237±0.007 | 1.370±0.008 |
| *SLC1A6* | 1.249±0.010 | 1.316±0.046 |
| *SLC1A7* | 0.872±0.115 | 1.411±0.013 |
| *SLC38A1* | 1.084±0.008 | 0.946±0.118 |
| *SLC38A2* | 0.984±0.003 | 0.981±0.004 |
| *SLC38A4* | 1.182±0.005 | 1.066±0.003 |
| *SLC16A10* | 1.134±0.005 | 1.145±0.004 |
| *SLC2A1* | 0.846±0.003 | 0.854±0.004 |
| *SLC2A3* | 0.981±0.004 | 0.976±0.011 |
| *SLC2A4* | 1.303±0.007 | 1.142±0.005 |
| *SLC2A9* | 1.129±0.003 | 1.019±0.005 |
| *SLC27A1* | 1.361±0.006 | 1.470±0.005 |
| *SLC27A2* | 1.022±0.003 | 0.973±0.004 |
| *SLC27A3* | 1.099±0.004 | 1.068±0.004 |
| *SLC27A4* | 1.094±0.003 | 1.114±0.005 |
| *SLC27A6* | 1.142±0.006 | 1.367±0.113 |
| *GOT2* | 1.186±0.088 | 1.398±0.006 |
| *CD36* | 0.973±0.005 | 0.902±0.004 |
| *MSR1* | 1.175±0.007 | 1.131±0.004 |
| *SCARB1* | 1.012±0.005 | 0.998±0.003 |
| *LDLR* | 1.021±0.004 | 1.016±0.004 |
| *VLDLR* | 1.130±0.005 | 1.033±0.076 |
| *ABCA1* | 1.122±0.006 | 1.129±0.005 |
| *ABCG1* | 1.195±0.005 | 1.183±0.004 |

Supplementary Method 1, Cotinine measurement by LC-MS/MS

**Chemicals**

All chemicals from Sigma-Aldrich except LC-MS grade methanol (VWR) and D3-cotinine (QMX Labs Ltd).

**Determination of cotinine in human liver and placenta**

Placenta and liver cotinine levels were quantified using a LC-MS/MS method as follows.

Cotinine and the internal standard (IS) ^2^H_3_-cotinine were dissolved in methanol at a concentration of 1 mg/mL and stored in aliquots at -20°C. Daily, the cotinine stock was diluted in 0.1% formic acid in water to give calibration standards in the range 0.1 – 30 ng/mL. The IS was diluted in 0.1% formic acid in water at a concentration of 1.2 µg/mL. Quality control samples were prepared in 0.1% formic acid in water at 0.25, 15 and 25 ng/mL cotinine and stored at -70°C.

Tissue samples were weighed and 1 mL 50/50/0.1 water/methanol/formic acid added along with 10 µL IS (12 ng). Samples were homogenised for 30 seconds using an Ultra-Turrax tissue disintegrator and vortexed mixed for a further 10 seconds. Following centrifugation at 4500 rpm for 5 minutes, the supernatant was applied to BondElut Plexa PCX cartridges (60 mg/3 mL, Crawford Scientific, UK) that had been pre-conditioned and equilibrated using 1.0 mL of methanol and 1.0 mL of 0.1% formic acid in water. The cartridges were washed with 1.0 mL 0.1% formic acid in water followed by 2 x 1.0 mL 95/5 methanol/0.1% formic acid in water and cotinine and the IS eluted with 1.0 mL 95/5 methanol/ammonium hydroxide. The eluate was evaporated to dryness under nitrogen at room temperature and the residue re-suspended in 100 µL 50/50/0.1 water/methanol/formic acid. After centrifugation at 14800 rpm for 5 minutes, 5 µL of the supernatant was injected onto the chromatograph. Standards and QC samples (1 mL) were subjected to extraction on BondElut Plexa PCX, evaporation and reconstitution as described.

Chromatography was performed on a Thermo Surveyor (Thermo Scientific, UK) system using a 150 x 2.1 mm ACE 3µ C18-AR column (Hichrom, UK) maintained at 50°C. The mobile phase consisted of 0.1% ammonium acetate (A) and methanol (B) and elution achieved with a linear gradient over 3 minutes from 10 to 100% B with a hold of 1 minute at 100% B. The flow rate was 200 µL/min and the samples were maintained at 4°C in the autosampler. Total run time was 8 minutes.

A Thermo TSQ Quantum triple quadrupole mass spectrometer was used in positive electrospray ionisation mode for the detection of cotinine. Quantification was performed using single reaction monitoring (SRM) scan mode using the following transitions: cotinine m/z 177.0 – 80.1 and ^2^H_3_-cotinine m/z 180.0 – 80.1. Flow injection analysis was used to optimise the MS/MS conditions as follows: spray voltage 4000 V, sheath gas pressure 60, auxiliary gas pressure 0, capillary temperature 375 °C, skimmer offset -10 V, collision pressure 1.7 mTorr and collision energy 25V.

Instrument control and peak integration and quantification were performed using Thermo Xcalibur software (v. 2.0.7 SP1). Weighted least squares linear regression with a weighting factor of 1/X was used to quantify the cotinine concentration in unknown samples by comparing peak area ratios (analyte/IS) with those obtained from a multi-level calibration standard curve.


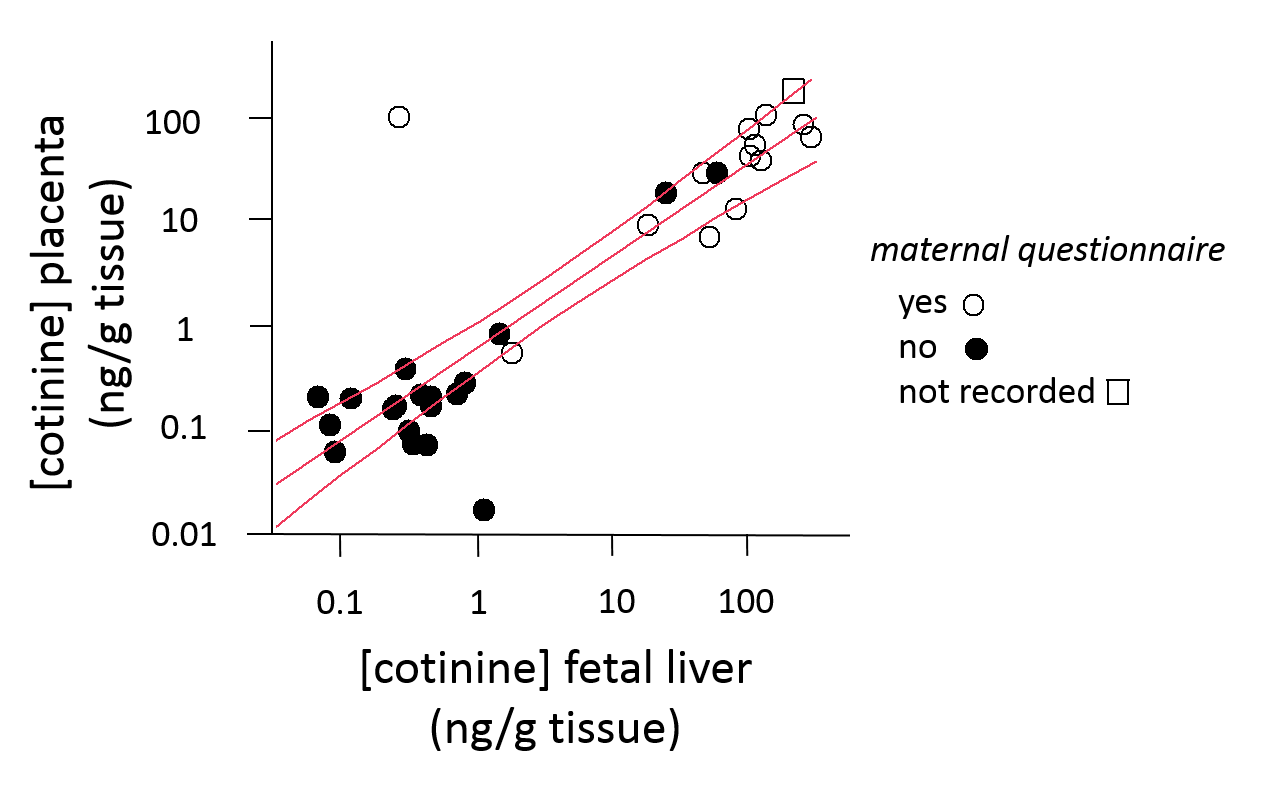


**Figure S1. Close agreement between placental and fetal liver cotinine concentrations in 35 electively terminated pregnancies.** Cotinine concentrations between fetal liver and placenta were highly correlated (R squared=0.82), with the exception of a single sample (LC-MS measurement repeated with same result). Values normalized to weight of tissue. Two samples logged as non-smokers according to maternal questionnaire however had high levels of cotinine, which may represent second-hand smoke exposure. In our statistical analyses these fetuses were classed as smoking-exposed.


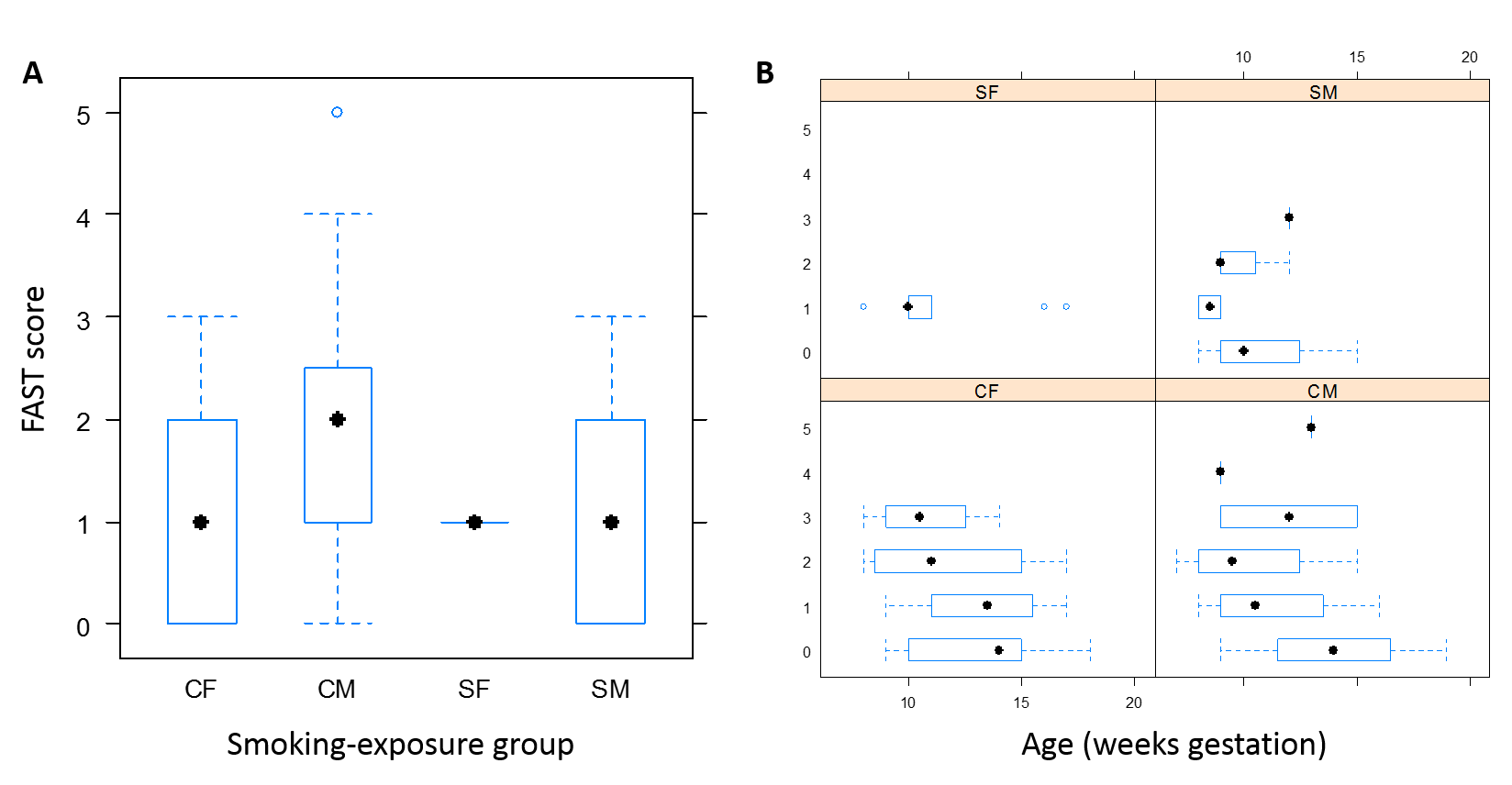


**FIGURE S2. Fast Alcohol Screening Test (FAST) score distributions. A)** FAST scores are not significantly different among the 4 exposure groups. **B)** FAST scores are unevenly spread across fetal age (weeks gestation) in this placenta-liver pair cohort. CF control female; CM control male; SF smoking-exposed female, SM smoking-exposed male.


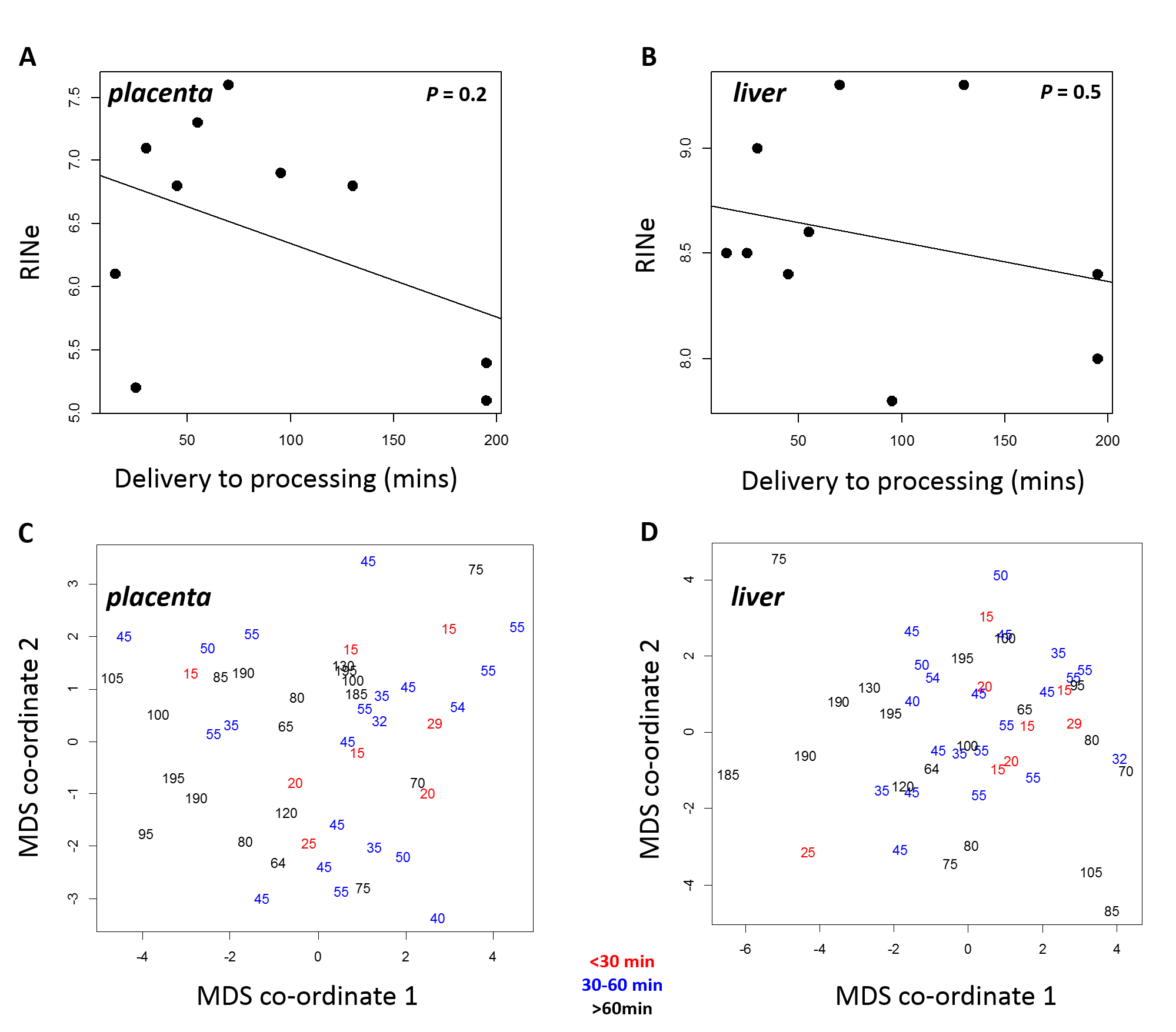


**Figure S3. RNA quality does not correlate with minutes elapsed from delivery to tissue processing. A)** Placental RNA quality score against delay to processing, no correlation (*P*=0.2). **B)** Liver RNA quality score against delay to processing, no correlation (*P*=0.5). Both n=10 from same pregnancy, randomly selected from SAFeR cohort. RINe: RNA integrity number. **Multi-Dimensional Scaling (MDS) plots** of all **C)** placental and **D)** liver transcripts measured by RT qPCR to detect grouping according to varying processing times (n=51, from the current study cohort). Red points: samples collected within 30 minutes of delivery; blue points: 30-60 minutes and; black points: over 60 minutes.


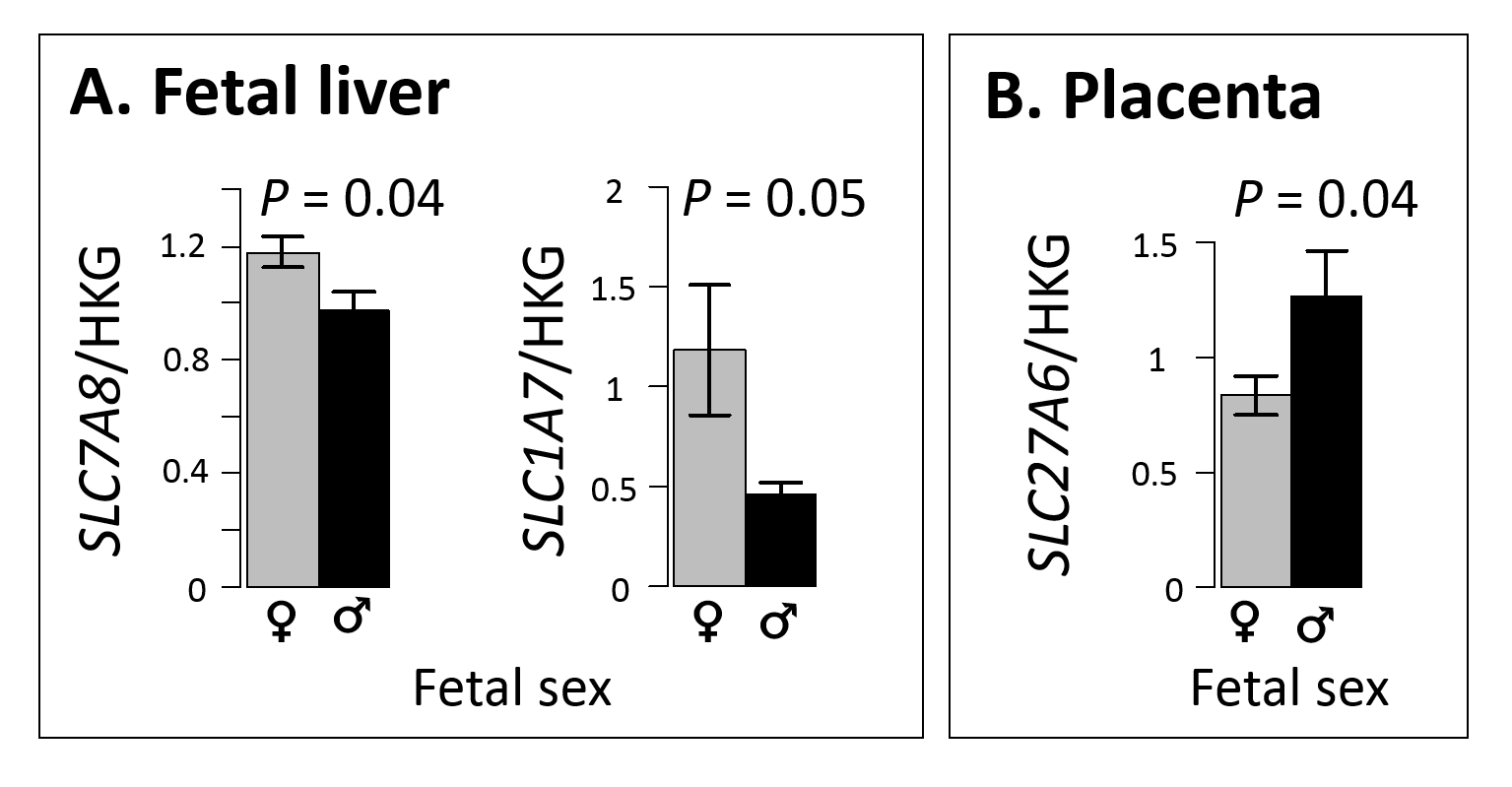


**FIGURE S4. Fetal sex specific changes in mean transporter transcript expression levels.** Sex-specific differences in mean transcript expression levels irrespective of gestational age were only seen for **A)** *SLC7A8* and *SLC1A7* in the fetal liver and **B)** *SLC27A6* in the placenta. All data was normalised to *SDHA*. Error bars mean±SEM, n=54 placenta-liver pairs, per group: n=25 males and n=29 females.
